# Quality of Dying in Nursing Home Residents Dying with Dementia: Does Advanced Care Planning Matter? A Nationwide Postmortem Study

**DOI:** 10.1371/journal.pone.0091130

**Published:** 2014-03-10

**Authors:** An Vandervoort, Dirk Houttekier, Robert Vander Stichele, Jenny T. van der Steen, Lieve Van den Block

**Affiliations:** 1 End-of-Life Care Research Group, Vrije Universiteit Brussel (VUB) & Ghent University, Brussels, Belgium; 2 Heymans Institute of Pharmacology, Ghent University, Ghent, Belgium; 3 EMGO Institute for Health and Care Research and Expertise Center for Palliative Care, Department of General Practice & Elderly Care Medicine, VU University Medical Center, Amsterdam, The Netherlands; 4 Department of Family Medicine, Vrije Universiteit Brussel, Brussels, Belgium; Iranian Institute for Health Sciences Research, ACECR, Iran (Republic of Islamic)

## Abstract

**Background:**

Advance care planning is considered a central component of good quality palliative care and especially relevant for people who lose the capacity to make decisions at the end of life, which is the case for many nursing home residents with dementia. We set out to investigate to what extent (1) advance care planning in the form of written advance patient directives and verbal communication with patient and/or relatives about future care and (2) the existence of written advance general practitioner orders are related to the quality of dying of nursing home residents with dementia.

**Methods:**

Cross-sectional study of deaths (2010) using random cluster-sampling. Representative sample of nursing homes in Flanders, Belgium. Deaths of residents with dementia in a three-month period were reported; for each the nurse most involved in care, GP and closest relative completed structured questionnaires.

**Findings:**

We identified 101 deaths of residents with dementia in 69 nursing homes (58% response rate). A written advance patient directive was present for 17.5%, GP-orders for 56.7%. Controlling for socio-demographic/clinical characteristics in multivariate regression analyses, chances of having a higher mean rating of emotional well-being (less fear and anxiety) on the Comfort Assessment in Dying with Dementia scale were three times higher with a written advance patient directive and more specifically when having a do-not-resuscitate order (AOR 3.45; CI,1.1–11) than for those without either (AOR 2.99; CI,1.1–8.3). We found no association between verbal communication or having a GP order and quality of dying.

**Conclusion:**

For nursing home residents with dementia there is a strong association between having a written advance directive and quality of dying. Where wishes are written, relatives report lower levels of emotional distress at the end of life. These results underpin the importance of advance care planning for people with dementia and beginning this process as early as possible.

## Introduction

Advance care planning (ACP) is considered a central component of good quality palliative care and is especially relevant for people who lose the capacity to make decisions at the end of life, which is the case for many nursing home residents with dementia[1]–[3]. ACP is a communication process between the patient and his/her care providers, which may involve family or friends, about the goals and desired direction of care at the end of life in the event of loss of capacity to make decisions [4], [5]. The process of ACP can result in the making of advance directives in writing, often in the form of life-limiting treatment decisions [6], [7]. When planning for current and future care, general practitioner (GP) orders are also very common among nursing home residents dying with dementia. In a recent study in Belgium, we found that GP orders consisting of instructions from the GP placed in the resident’s medical file governing the use of specific treatments toward the end of life were present in 59% of cases [6].

The advance planning of care can be of particular importance for nursing home residents with dementia considering the loss of decision-making capacity inherent to the disease [2], [8]–[14]. However the beneficial impact on outcomes of ACP – verbal ie spoken or in writing – and of GP orders on the quality of dying is unclear, especially for this disease population. Earlier studies concerning ACP are mostly limited to other patient populations [15] or to studying written advance patient directives only and suggest that advance directives have a low potential for improving patient care [16]–[19]. Some recent studies do suggest that ACP is associated with the greater use of hospices, less use of life-sustaining treatments such as feeding tubes, lower likelihood of terminal hospitalization, fewer concerns with physician communication and the resident being better informed about the dying process. However, these studies did not focus on residents with dementia [1], [9], [20]–[22]. One previous study in Belgium did study nursing home residents with dementia and found that they were less likely to die in hospital if a GP order was present [6]. However, existing research mainly focused on care processes or care utilization and its association with ACP rather than relating different forms of ACP to patient outcomes and quality of dying.

In this study, we investigate to what extent ACP in the form of written advance directives, verbal ie spoken advance communication with patient and/or relatives and the existence of GP orders relate to the quality of dying in nursing home residents with dementia.

## Methods

### Ethics Statement

The research procedures respected privacy/confidentiality of patients and respondents. The study protocol was approved by the Medical Ethics Committee of UZ Brussel (University Hospital of Brussels).

### Design

We performed a cross-sectional study of deaths to describe the end of life of nursing home residents dying with dementia, representative for Flanders, Belgium (Dying Well with Dementia study) [2]. All deaths from a random sample of Flemish nursing homes were recorded in 2010, stratifying homes by region (five provinces) and subsequently by bed capacity (up to 90 or more than 90) and ownership (public, private/nonprofit, private/profit).

Questionnaires were completed by the nurse most involved in the resident’s care, the resident’s general practitioner (GP), a family member or friend closely involved in the resident's care, and the nursing home administrator in the case of nursing home residents.

### Setting and Selection of Residents

Nursing home residents with dementia who had died in the last three months were identified from all nursing home deaths using a two-stage screening protocol:

1. In the first step residents were included if they met the criteria for (1) ‘category C dementia’ ie ‘being completely care dependent or needing help for bathing, dressing, eating, toileting, continence and transferring plus being disoriented in time and space’, OR (2) disorientation in time and space (KATZ scale ≥3 or having ‘almost daily a problem with disorientation in time and space’) [23].
2. In the second step additional eligibility criteria required that the family physician or nurse indicated that the resident ‘had dementia’ or ‘was diagnosed with dementia’.

### Measurements

The after-death questionnaires sent to nurses surveyed the socio-demographic characteristics of the person: their health status, care planning and advance care planning communication. Those sent to the relatives used the Comfort Assessment in Dying with Dementia (CAD-EOLD) to measure the quality of dying.

### Content of After-death Questionnaires

DEMOGRAPHICS AND CHARACTERISTICS OF RESIDENTS (by nursing home administrator).

- age, gender
- length of nursing home stay
- whether the residents lived in a special care unit for dementia (ie a closed unit within the nursing home where care is adjusted to the special needs of residents with dementia, sometimes with staff trained in dementia care)
- place of death

HEALTH STATUS.

- median survival time after onset of dementia (by the nurse)
- Bedford Alzheimer Nursing Severity-Scale (BANS-S) [24] (by the nurse)
- co-existing conditions (by the GP)
- level of dementia (by nurse and/or GP)

DOCUMENTED Care planning (by the nurse).

- presence of written advance patient directives
- existence of proxy authorized in writing to take decisions about treatment in the case of loss of capacity
- presence of GP orders (documentation in the resident’s file of GP orders limiting the use of specific treatments at the end of life).

These documents considered the following treatment decisions: do-not-hospitalize, do-not-resuscitate, do-not-intubate, the withholding/withdrawing of artificial food and/or fluids, withholding/withdrawing other treatments, withholding/withdrawing antibiotics, euthanasia (ie administration of a lethal drug at the explicit request of the resident), terminal sedation (ie using medication to keep the resident unconscious until death), trying all life-prolonging treatments, other.

ACP COMMUNICATION about a resident’s future care (by the nurse).

- resident expressed wishes to nurse concerning medical treatment at end of life
- nurse spoke with resident concerning medical treatment and the desired goals of care in the last phase of life
- nurse spoke with family member or friend in advance concerning medical treatment and the goals of care in the last phase of life

Comfort Assessment in Dying with Dementia (CAD-EOLD, by the relative) [25].

Evaluates the comfort around dying of cognitively impaired persons (three response options 1–3). The total score ranges from 14 to 42 with higher scores indicating better comfort. The four subscales and 14 individual items are:

- Physical Distress: discomfort, pain, restlessness, and shortness of breath
- Dying Symptoms: shortness of breath, choking, gurgling, and difficulty swallowing
- Emotional Distress: fear, anxiety, crying, and moaning
- Well Being: serenity, peace, and calm

For Physical Distress, Dying Symptoms and Emotional Distress the range is from 4 (worst) to 12 (best); for Well-Being from 3 (worst) to 9 (best).

### Analyses

Analyses were performed with PASW statistical software, 17.0 (SPSS Inc., Chicago, IL). The CAD-EOLD total score is calculated by summing the value of each item. Missing CAD-EOLD items were imputed with the overall residents’ mean for that item in cases where there were four or fewer missing scores on the scale. Coding was reversed when applicable such that higher scores represent more comfort.

Descriptive results are presented in frequency tables. Bivariate associations were tested using Mann-Whitney U test (because of the ordinal measurements of the CAD-EOLD items, significance level p<.05,). We performed a multivariate ordinal regression (because of non-normal distribution of the residuals) to explore characteristics ie care planning and advance care planning communication (from the nurse) associated with the outcome measure ie Comfort Assessment in Dying with Dementia (CAD-EOLD, from the relative). For the multivariate ordinal regression analyses the CAD-EOLD total score (dependent variable at ordinal level) was categorized into five categories and the subscales scores into four categories based on distribution (reference = highest scores/category or better comfort) and ACP as independent variable (dichotomous, yes or no, reference = no) controlled for age, gender, level of dementia and sentinel events.

## Results

### Sample Description

Sixty nine nursing homes (58% response rate) participated, representative of all nursing homes in Flanders. Of the 477 deceased nursing home residents, 205 had dementia at the time of death; 15 cases were excluded because there was no relative available [2]. Response rates for questionnaires were nurses 88.4%, GPs 52.9%, nursing home administrators 95.0% and relatives 53.2% [2]. Cases were selected for analysis when both the nurse and the relative questionnaires were completed (n = 101). The median time between death of the resident and receipt of the questionnaire was 65 days for nurses (interquartile range 37–91 days), 82 days for GPs (IQR 48–137 days) and 134 days for relatives (IQR 45–104 days). The realized sample of nursing home residents was representative for age (p-value = 0.55) for the population of nursing home residents dying with dementia insured by the two largest insurance companies in Flanders covering more than 70% of the population (analysis not in table). Men were overrepresented in our sample (p = .02). Non-response analysis showed no differences for important resident (age, gender, length of stay, place of death, cognitive status, disease severity) and care (end-of-life treatments, nursing care) characteristics between residents of participating and non-participating GP [2].

At time of death, 51% had very severe or advanced dementia, 25% severe dementia and 24% moderate or mild dementia. Sixty three percent of all residents with dementia were aged 85 or older and 42% were male (Table 1). The median length of stay was 2.1 years. The mean BANS-S score was 20.7 (range from 7 to 28; higher scores indicate greater functional and cognitive disability). The most common co-existing conditions were cardiovascular (41%) and neurological (19%). Place of death was the nursing home in 92%.

**Table 1:** Deceased Nursing Home Residents with Dementia in Flanders, Belgium: Description of the Sample (N = 101).*.

RESIDENT CHARACTERISTICS	N (%)
Age – yr	
<80	16 (16)
80–84	20 (20)
85–89	26 (27)
90–94	21 (21)
≥95	15 (15)
Gender, male	40 (42)
Median (quartiles) length of nursing home stay (years)	2.1 (1.0–3.7)
Living in special care unit for dementia	50 (53)
Place of death	
Nursing home	88 (92)
General hospital ward or intensive care unit	6 (6)
Palliative care unit	2 (2)
HEALTH STATUS	
BANS-S one month before death (mean ± SD) †	20.7±3.7
Co-existing conditions ‡	
Malignant tumour	7 (10)
Cardiovascular	28 (41)
Respiratory	9 (13)
Neurological	13 (19)
Kidney disease	7 (10)
Other	12 (17)
None of the above	13 (19)
Level of dementia	
Moderate or mild dementia	24 (24)
Severe dementia	25 (25)
Very severe or advanced dementia	52 (51)
QUALITY OF DYING ACCORDING TO THE RELATIVE §	mean ± SD
CAD-EOLD total scores II	29.6±6.4
CAD-EOLD subscales	
Physical Distress	8.3±2.3
Dying Symptoms	8.1±2.6
Emotional Distress	9.2±2.3
Well-Being	6.0±1.8
CAD-EOLD individual items	
Discomfort	2.2±0.7
Pain	2.0±0.8
Restlessness	1.9±0.8
Shortness of breath	2.2±0.9
Choking	2.1±0.8
Gurgling	2.1±0.8
Difficulty swallowing	1.8±0.8
Fear	2.2±0.8
Anxiety	2.1±0.8
Crying	2.6±0.6
Moaning	2.3±0.8
Serenity	2.0±0.7
Peace	2.0±0.6
Calm	1.9±0.7

*Missing values are for age n = 3, for gender n = 6, LOS n = 5, living in care unit for dementia n = 6, BANS-S n = 4, co-existing conditions n = 32 (of which 31 because no questionnaire was received from the GP), CAD-EOLD n = 9.

† Scores on the BANS-S (Bedford Alzheimer Nursing Severity Scale) range from 7 to 28; higher scores indicate greater functional and cognitive disability.

‡ Multiple answers possible.

§ CAD-EOLD. All items were (re)coded so that higher scores means better symptoms management.

II The CAD-EOLD total score is constructed by summing the value of each item. It ranges from 14 to 42 with higher scores indicating better symptom control.

The mean CAD-EOLD total score was 29.6 (Table 1). CAD-EOLD subscales scores (mean) for Physical Distress were 8.3, Dying Symptoms 8.1, Emotional Distress 9.2 and Well-Being 6.0.

### Frequency of ACP

A written advance patient directive was present in 17.5% of cases (Table 2). GP orders were reported in 56.7% and they were discussed with the resident in 3.2%. The nurse spoke with the residents concerning medical treatments and the desired goals of care in the last phase of life in 13.7% of cases (Table 3).

**Table 2:** Association between documented care planning and quality of dying (CAD-EOLD total score and subscales) among nursing home residents dying with dementia in Flanders, Belgium (n = 101).

	N (%)*	Comfort Assessment in Dying with Dementia (CAD-EOLD) measured by the resident’s relative									
		TOTAL SCORE		SUBSCALES SCORES							
				Physical Distress		Dying Symptoms		Emotional Distress		Well-Being	
		Mean (SD) †	AOR ‡	Mean (SD) †	AOR ‡	Mean (SD) †	AOR ‡	Mean (SD) †	AOR ‡	Mean (SD) †	AOR ‡
RESIDENT’S ADVANCE CARE PLANNING											
Written advance patient directive,Yes	17 (17.5)	31.9 (7.1)	ns	8.8 (2.9)	ns	8.6 (2.4)	ns	10.2 (2.3)	2.99 [1.1–8.3]	6.2 (1.9)	ns
No	80 (82.5)	29.1 (6.3)		8.2 (2.2)		8.0 (2.6)		9.0 (2.3)		5.9 (1.9)	
Do-not-hospitalise,Yes	14 (14.4)	32.8 (6.4)	ns	8.8 (2.9)	ns	9.1 (2.2)	ns	10.4 (1.8)	2.54 [0.8–7.7]	6.4 (2.0)	ns
No	83 (85.6)	29.1 (6.4)		8.2 (2.2)		8.0 (2.6)		9.0 (2.4)		5.9 (1.9)	
Do-not resuscitate,Yes	13 (13.4)	32.9 (5.6)	ns	9.3 (2.7)	ns	8.6 (2.3)	ns	10.6 (1.7)	3.45 [1.1–11]	6.5 (1.6)	ns
No	84 (86.6)	29.1 (6.5)		8.1 (2.2)		8.1 (2.7)		9.0 (2.4)		5.9 (1.9)	
Proxy decision-maker assigned,Yes	5 (5.7)	29.1 (7.6)	ns	8.2 (2.6)	ns	7.8 (3.0)	ns	9.4 (3.3)	ns	5.6 (0.9)	ns
No	82 (94.3)	29.4 (6.3)		8.1 (2.3)		8.0 (2.6)		9.1 (2.3)		5.9 (1.9)	
GENERAL PRACTITIONER’S ORDERS (GP ORDERS)											
GP orders,Yes	55 (56.7)	29.0 (6.1)	ns	8.3 (2.2)	ns	7.9 (2.6)	ns	9.0 (2.4)	ns	5.9 (1.9)	ns
No	42 (43.3)	30.6 (7.1)		8.3 (2.4)		8.5 (2.6)		9.4 (2.3)		6.1 (1.9)	
GP-orders were discussed with Resident,Yes	2 (3.2)	35.2 (4.0)	ns	10.5 (0.7)	ns	10.0 (2.8)	ns	11.0 (1.4)	ns	6.0 (0.0)	ns
No	61 (96.8)	29.2 (6.3)		8.2 (2.3)		8.0 (2.6)		9.1 (2.4)		6.0 (1.9)	

*Numbers of categories of variables may not add up to 101 because of missing values.

† The CAD-EOLD total score ranges from 14 to 42 with higher scores indicating better symptom control. The CAD-EOLD subscales range for Physical Distress, Dying Symptoms and Emotional Distress from 4 (worst) to 12 (best). For Well-Being it ranges from 3 (worst) to 9 (best).

‡ Adjusted odds ratio using a multivariate ordinal regression model.

**Table 3:** Association between ACP communication and quality of dying (CAD-EOLD total score and subscales) among nursing home residents dying with dementia in Flanders, Belgium (n = 101).

	N (%)*	Comfort Assessment in Dying with Dementia (CAD-EOLD) measured by the resident’s relative									
		TOTAL SCORE		SUBSCALES SCORES							
				Physical Distress		Dying Symptoms		Emotional Distress		Well-Being	
		Mean (SD) †	AOR ‡	Mean (SD) †	AOR ‡	Mean (SD) †	AOR ‡	Mean (SD) †	AOR ‡	Mean (SD) †	AOR ‡
RESIDENT’S ADVANCE CARE PLANNING, VERBALLY											
Resident expressed wishes to nurse concerning medical treatments at end-of-life,Yes	13 (17.1)	32.1 (8.0)	ns	8.0 (2.9)	ns	8.4 (3.1)	ns	9.3 (3.1)	ns	7.0 (2.0)	ns
No	63 (82.9)	29.1 (6.4)		8.2 (2.2)		8.1 (2.7)		9.0 (2.2)		5.8 (1.8)	
Nurse spoke with resident concerning medical treatments and the desired direction of care in the lastphase of life,Yes	13 (13.7)	32.8 (5.1)	ns	8.6 (2.8)	ns	8.6 (2.5)	ns	9.7 (2.6)	ns	6.9 (1.4)	ns
No	82 (86.3)	29.3 (6.7)		8.2 (2.3)		8.1 (2.6)		9.2 (2.3)		5.9 (1.9)	
COMMUNICATION WITH RELATIVES											
Nurse spoke with family member or friend in advance concerning medical treatments and the desireddirection of care in the last phase of life,Yes	57 (60.6)	28.5 (6.8)	1.21[0.3–5.6]	7.8 (2.3)	0.28[0.08–0.98]	7.5 (2.6)	0.26 [0.1–0.6]	8.9 (2.5)	ns	6.0 (1.9)	ns
No	37 (39.4)	31.4 (6.0)		9.0 (2.2)		9.0 (2.5)		9.6 (2.1)		5.9 (1.9)	

*Numbers of categories of variables may not add up to 101 because of missing values.

† The CAD-EOLD total score ranges from 14 to 42 with higher scores indicating better symptom control. The CAD-EOLD subscales range for Physical Distress, Dying Symptoms and Emotional Distress from 4 (worst) to 12 (best). For Well-Being it ranges from 3 (worst) to 9 (best).

‡ Adjusted odds ratio using a multivariate ordinal regression model.

### Association between Care Planning and Quality of Dying

In tables 2 and 3 the results of multivariate ordinal regression analyses are reported, showing associations between various forms of care planning and quality of dying in the last week of life (using the CAD-EOLD) as reported by the resident’s relative. The chances of having a higher ie better rating on the Emotional Distress subscale were three times greater for residents with a written advance patient directive than for those without such a directive (Table 2, Adjusted OR 2.99; CI, 1.1–8.3). A do-not-resuscitate order decreased the chance of experiencing Emotional Distress in the last week of life compared with not having a do-not-resuscitate order (Adjusted OR 3.45; CI, 1.1–11). Bivariate analyses showed that having a do-not-hospitalize order was associated significantly with experiencing less emotional distress (p-value = 0.038; analysis not shown in table), however this did not remain significant in multivariate analyses (Adjusted OR 2.54; CI, 0.8–7.7).

Studying the individual items of the Emotional Distress subscale of the CAD-EOLD (analysis not shown in table) showed that having a written advance directive decreased the chance of experiencing fear in the last week of life compared with not having a written advance directive (AOR 3.72; CI, 1.2–11.8) and that having a do-not-resuscitate order decreased the chance of experiencing fear (AOR 3.85; CI, 1.1–13.4) and anxiety (AOR 3.57; CI, 1.0–12.3).

The quality of dying was not significantly associated with the existence of a GP order (Table 2).

No association was found between the quality of dying as judged by the relative and verbal communication such as the resident expressing their wishes to the nurse or the nurse speaking with the resident concerning medical treatments at the end of life or the desired direction of care (Table 3).

Residents about whom the nurse had spoken with a family member or friend in advance concerning the desired direction of care were more likely to have a lower rating on Physical Distress subscale (Adjusted OR 0.28; CI, 0.08–0.98) and Dying Symptoms subscale of the CAD-EOLD (Adjusted OR 0.26: CI, 0.1–0.6) (Table 3).

Studying the relationships between the individual item scores of the CAD-EOLD scale and care planning (not shown in tables) shows that residents about whom the nurse had spoken with a family member or friend in advance were also more likely to have lower ratings of discomfort (Physical Distress subscale, AOR 0.23; CI, 0.09–0.60), restlessness (Physical Distress subscale, AOR 0.41; CI, 0.17–0.98), gurgling (Dying Symptoms subscale, OAR 0.32; CI, 0.1–0.8) and difficulty swallowing (Dying Symptoms subscale, AOR 0.24; CI, 0.1–0.6).

## Discussion

In this study among nursing home residents dying with dementia we found a strong association between the existence of a written advance directive and the quality of dying, in particular with lower levels of emotional distress ie fear and anxiety. No association was found between there having been spoken communication between the care staff and resident or the existence of a GP order and the quality of dying, while spoken communication between a nurse and a relative appears to have a negative association with the quality of dying.

A major finding is that nursing home residents with dementia who had a written advance directive were judged by relatives to have had higher ratings of emotional wellbeing while dying than those who had no written advance directive; specifically, they were judged to have shown less fear and anxiety while dying. This is a striking finding considering that previous studies mainly show the association between advance care planning and the use of end-of-life care (eg more hospice enrolment, less aggressive treatment), place of death or relatives’ satisfaction with care [1], [20], [21] While this study relates the existence of written advance directives to a better quality of dying, the fact that advance directives may affect emotional rather than physical distress or dying symptoms (eg pain or shortness of breath) is intriguing. Although we cannot disentangle the particular mechanisms responsible for this association, a number of hypotheses related to the resident him/herself, the relative, nurse or the nursing home may be formulated. Firstly, advance directives might be the result of a psychological process by the resident, induced by a thorough process of communication concerning their preferred care at the end of life, ultimately resulting in dying with less fear and anxiety. Considering that half of residents dying with dementia are not in an advanced stage a month before dying [2], this seems a possible explanation. Secondly, the scores of the relatives might reflect their own emotional state of mind more than the actual situation of the resident. On the one hand, this might suggest that in cases where an advance directive was present, relatives interpret the condition of the resident as emotionally better. However, a previous report on prevalence of ACP in this population shows that relatives are not always aware of the existence of advance directives reported by the nurses [7]. On the other hand, nurses might have communicated with relatives about the dying process of the resident, positively interpreting their emotional state where an advance directive is present, reassuring the relatives about how the resident is dying. However, if this hypothesis were true, it is surprising that the items measuring well-being such as serenity or peace were not associated. Thirdly, some nursing homes might have an established palliative care culture where advance care planning is performed extensively which might be associated with more patients dying well. This seems less likely however considering that we found no analogous association between advance directives and physical distress or dying symptoms.

Regardless of the precise reasons or cause-effect hypotheses, the results do show the strong relationship between patient-centered planning and quality of dying which should be unravelled in more detail in further research. Considering the relatively low prevalence of advance care planning and advance directives, the results advocate the need to increase early communication about end-of-life issues for people with dementia in nursing homes, enabling them to reflect on their options and facilitating a psychological process oriented towards the final period of life.

One relationship found in our study is difficult to explain ie that verbal communication between the nurse and a relative showed a negative association with the quality of dying (discomfort, restlessness, gurgling, difficulty swallowing). One possible explanation is that these conversations take place reactively or ad hoc – when residents are experiencing clinical complications at the end of life ^[2]^ – ie too late in the illness trajectory. Ideally, advance care planning starts in the earlier stages of the disease, especially for nursing home residents with dementia, and focuses on both current and future care. This would imply the benefits of a proactive attitude among nursing home staff in developing advance care plans and facilitating end of life discussion with all involved in care [29].

It is interesting to note that neither discussion between residents and nurses nor the presence of GP orders regulating end-of-life treatment decisions – which both occurred considerably more often than written advance directives – were strongly related to the quality of dying (ie the differences were not large enough to reach significance). One possible explanation is that the conversations between resident and nurses were limited in frequency, not repeated over time or not intense or thorough enough, and that the GP orders were made without involving the resident him/herself which our previous study in Belgium has shown is often the case. Hence, for this specific patient population of nursing home residents with dementia, our results seem to suggest that having a conversation about future care might not be associated with a better quality of dying if not resulting in a clear formulation of wishes in writing. This of course does not suggest that merely presenting people with paper forms in which they need to state their preferences for end-of-life treatments, without careful or regular conversations about future options, will achieve better results; other studies have shown that focusing on the completion of advance directives alone does not improve medical end-of-life care [1], [19].

This is the first study to relate ACP to the quality of dying of nursing home residents with dementia in Flanders. A strength of the study is the use of data from a large representative nationwide population-based study and the identification of a clear study population (deceased nursing home residents with dementia). Other strengths contributing to the quality of the data are the high response rates (the lowest were from GPs (52.9%) and relatives (53.2%), although these are still higher than average for physician postal surveys and for most medical surveys in Belgium [26], [27]), the use of a two-step screening protocol to identify the study population and the use of a validated scale (CAD-EOLD) to measure the quality of dying [28]. A non-response analysis showed no differences between residents whose family physicians did or did not respond on important characteristics such as demographics, cognition, decision-making capacity, treatment and care [2]. Finally, we were able to measure residents’ outcomes from the perspective of the relative while care processes were reported by nurses.

Nevertheless, the study has several limitations. It was retrospective, therefore memory bias cannot be ruled out, although this was limited by focusing on the final week of life. Also, when interpreting the results, we acknowledge that were relying on relatives’ and nurses’ reports and perception of care planning and quality of dying, which may differ from what residents themselves would have reported [7], [15]. Also, by using a cross-sectional design we can only establish associations and not causal relationships.

In conclusion, our study shows a clear relationship between having expressed end-of-life care treatment preferences in a written patient advance directive and dying with less emotional distress. Regardless of the specific mechanisms behind this relationship and the respective roles of relatives, residents and caregivers, these results underline the importance of beginning the process of ACP for people with dementia as early as possible, and the need to increase this practice.
